# Application of dimensionality reduction and clustering techniques for the analysis of Carrion's disease cases in the period 2000–2024

**DOI:** 10.3389/frai.2026.1883357

**Published:** 2026-08-11

**Authors:** Moisés Evangelista Gamarra, Jerremi Aron Chancan Labajos, Pamela Estefani Figueroa Rosas, Ronaldo Edilberto Alvarez Manrique

**Affiliations:** Program of Software Engineering with Artificial Intelligence, Servicio Nacional de Adiestramiento en Trabajo Industrial (SENATI), Lima, Peru

**Keywords:** bartonellosis, Carrión's disease, clustering, public health, unsupervised learning

## Abstract

The heterogeneous geographic distribution and the complex dynamics of Carrion's disease challenge conventional epidemiological surveillance in Peru. To address this, this study applied unsupervised machine learning to 43,534 national records (2000–2024). Following a rigorous data cleaning process—which resolved duplicate records, missing information, and outliers using Tukey's interquartile range (IQR)—the dimensionality reduction approaches MCA and FAMD coupled with the K-Means algorithm were evaluated. The calibration of the Silhouette, Davies–Bouldin, and Calinski–Harabasz indices determined that the combination of FAMD and K-Means provided the best clustering quality, identifying two clearly differentiated epidemiological profiles. An ablation control experiment demonstrated that, even after excluding the ICD-10 diagnostic coding, the geometric structure of the clusters remained highly stable, indicating that the temporal, geographic, and demographic variables contain sufficient information to preserve the clustering structure. This internal consistency was indicated through *bootstrap* resampling simulations. In conclusion, the coupling of FAMD and K-Means establishes a stable and reproducible framework for advanced exploratory epidemiology, constituting a valuable complementary tool to support public health surveillance and guide strategic decision-making in public health.

## 1 Introduction

Neglected tropical diseases affect vulnerable communities in low- and middle-income countries, placing a substantial burden on the healthcare systems of these nations. For this reason, **Carrion's disease** constitutes a public health problem in some regions of Peru and Ecuador (Guimarães et al., 2025). Its mortality rate reaches up to 88% among untreated cases during the acute phase of the disease (Vargas et al., 2012), creating challenges for the diagnosis, surveillance, and control of the potential vectors of this disease, which has been particularly difficult to manage from both clinical and epidemiological perspectives because of the presence of two clearly differentiated stages: an acute phase and a chronic phase (Clemente et al., 2012).

The first phase, known as Oroya fever, produces severe acute hemolytic anemia. The second phase, referred to as Peruvian wart, is characterized by chronic angioproliferative skin lesions. This clinical complexity, together with the presence of asymptomatic carriers, the wide variety of clinical manifestations, adverse socioeconomic conditions in endemic areas, and limitations in diagnostic infrastructure, poses significant challenges for epidemiological surveillance systems (Augusto Tarazona et al., 2006; Enfermedad de Carrión (Bartonelosis) en el Perú, 2001).

More than a century after the disease was first described in 1885 by the Peruvian medical student Daniel Alcides Carrión, who deliberately inoculated himself with the infection to demonstrate the two phases of the disease, important gaps remain in the understanding of its transmission and manifestation patterns. Traditionally, these studies have been based on descriptive statistics, regression analysis, and passive surveillance methods, approaches that are insufficient to capture the complexity of the disease (Wang et al., 2019).

The recent evolution of artificial intelligence (AI) has reshaped the scope of epidemiological surveillance systems by strengthening their analytical capacity to track complex dynamics, predict outbreak spread, and provide public health with quantitative evidence for health crisis management. Within this new landscape, unsupervised machine learning has emerged as a valuable analytical approach; its value lies not in automating tasks but in its flexibility to recognize latent epidemiological structures within large institutional repositories. This data-driven diagnosis operates as a complementary approach, overcoming the methodological reductionism of linear tabulations and conventional descriptive analyses that often limit official reports (Mendes et al., 2025; Vargas-Santiago et al., 2025).

This highlights limitations related to data heterogeneity, the non-linear relationships between clinical and epidemiological variables, and the need to automatically identify subgroups of cases for risk characterization.

Within this context, this study aims to segment clinically significant and epidemiologically relevant subgroups from Carrion's disease records by comparing unsupervised machine learning algorithms applied to the national surveillance repository. Under this approach, the experimental design seeks to contribute to reducing the methodological gap that predominates in the current literature on this disease. The application of these machine learning techniques enables the identification of latent patterns and spatiotemporal dynamics that lie beyond the scope of conventional descriptive statistical tools.

## 2 Related work

### 2.1 Machine learning in infectious disease surveillance

The use of machine learning for the analysis of infectious diseases has gained increasing relevance in recent years due to the growing availability of health data and computational resources. These techniques can be applied to establish the foundation for the development of more effective strategies for managing this type of disease in the future, using metrics such as mean absolute error (MAE), true positive, true negative, false positive, and false negative rates, as well as derived metrics such as the area under the receiver operating characteristic curve (AUC-ROC), recall, and precision (Al Meslamani et al., 2024).

To determine whether a model is reliable, it is necessary to interpret its different performance indicators; however, this is a highly complex process, and it is not possible to determine individual confidence intervals for each prediction. Nevertheless, with current technology, it is possible to improve traditional epidemiological analysis, which relies on data exhibiting complex patterns that cannot be easily identified using conventional statistical methods.

Going beyond the traditional tasks of anomaly and outlier detection, unsupervised learning methodologies have begun to transform modern epidemiological surveillance platforms by uncovering clusters of cases that share specific clinical, demographic, and spatial characteristics. The application of these algorithms makes it possible to identify latent transmission patterns and characterize populations at risk that are exposed to complex health dynamics, operating as a supporting mechanism for the early detection of outbreaks and unusual increases in disease incidence. This analytical advantage is critical because the modeling process operates entirely without prior diagnostic labels or rigid institutional classifications to define cluster boundaries (Cheah et al., 2025; Akintola et al., 2025). This approach has been applied using several anomaly detection algorithms on malaria data from the Brazilian Amazon, employing methods such as PCA, SOS, and MCD, which achieved the highest coverage by detecting 73.53% of the anomalies, although this performance varied across regions (Eze et al., 2023).

The need to appropriately select and calibrate models for each epidemiological context makes it possible to establish more reproducible frameworks for new data. Early warning systems depend on this type of information because it facilitates outbreak identification and trend monitoring in endemic diseases. Algorithms have been developed for these systems that provide practical guidance for model selection according to data characteristics and surveillance objectives (Saab et al., 2025).

This is particularly relevant to the methodological framework of the present study, which focuses on neglected tropical diseases such as Carrion's disease.

### 2.2 Unsupervised learning for disease characterization

Unsupervised machine learning models have become established as critical tools in health records. Their integration into the healthcare ecosystem expands the scope of public health by providing stable methodologies for fine-grained patient stratification, the design of epidemiological surveillance alerts, and outbreak auditing.

The true value of these algorithms comes to the forefront when processing large observational repositories characterized by high multidimensionality and complex interactions between demographic variables and heterogeneous clinical manifestations that traditional statistical approaches do not fully capture (Idahor et al., 2025; Kim et al., 2025).

These models enable the identification of latent patterns in health data without relying on labeled datasets. Methods such as Latent Dirichlet Allocation (LDA) combined with the Poisson–Dirichlet Model (PDM) can be used to detect disease clusters and patient subgroups (Wang et al., 2019).

In the field of epidemiological surveillance, spatial partitioning and advanced segmentation techniques have been strategically applied to map and organize large-scale records of tuberculosis, malaria, COVID-19, and dengue, providing an additional analytical capability for identifying homogeneous population profiles and optimizing the strategic allocation of public health resources. Evidence reported in several studies demonstrates that the implementation of architectures such as K-Means, hierarchical clustering, and density-based approaches enables healthcare platforms to identify and characterize clinically relevant subgroups.

This methodological advantage is critical because the identified clusters encapsulate cross-cutting dynamics and risk profiles that remain invisible or diluted when analyzed using conventional descriptive statistical tools (Nalinthasnai et al., 2025; Silva et al., 2024).

Disease prediction can be performed across different datasets, representing a key step in the present study. Models such as DBSCAN (Density-Based Spatial Clustering of Applications with Noise) and Bayesian Gaussian Mixture Models demonstrate superior performance, although this varies according to the characteristics of the analyzed data. In particular, DBSCAN-based models achieve better performance on datasets in which disease classes are separated by density, whereas Bayesian Gaussian Mixture Models excel at detecting complex structures (Lu and Uddin, 2023).

In the analysis of Carrion's disease, the existence of different clinical phases may lead to variations in the results, thereby providing a better understanding of the overall epidemiological landscape and facilitating the selection of the most appropriate analytical strategy.

### 2.3 Bartonellosis and Carrión's disease: current state

This disease constitutes a public health problem in South America, particularly in Peru. Therefore, a systematic review of the literature on this topic provides guidance for establishing research priorities aimed at disease elimination, while also addressing critical knowledge gaps and improving the understanding of its transmission, vectors, and clinical management (Clemente et al., 2012).

The main challenges lie in its two clearly differentiated clinical phases: the acute hemolytic phase (**Oroya fever**), which is the most severe and exhibits high mortality rates, and the chronic eruptive phase (**Peruvian wart**), characterized by angiogenic skin lesions. Current clinical perspectives on the disease in Peru focus on the variability of these clinical phases and the challenges associated with its diagnosis and treatment (Vargas et al., 2012).

The technical guidelines issued by the **Ministerio de Salud** of Peru establish standardized protocols for the management and control of the disease. However, they lack optimized surveillance strategies and risk stratification approaches (Ministerio de Salud, 2025). In contrast, a variety of national public health surveillance platforms have begun to incorporate unsupervised learning, integrating these algorithms as a complementary tool to strengthen traditional syndromic surveillance frameworks and support the automated stratification of infectious disease cases. The application of this analytical framework highlights the feasibility and technical potential of such methodologies when addressing complex endemic settings. In these environments, where diseases exhibit highly heterogeneous biological behavior and transmission dynamics, advanced spatial partitioning serves as a critical tool for capturing cross-cutting population dynamics and risk profiles that are often overlooked by conventional descriptive approaches (First Workshop on AI Awareness for Threat Detection and Epidemic Intelligence, 2024; Geffroy et al., 2026).

Although artificial intelligence has rapidly expanded within the field of epidemiology, the majority of recent scientific research has focused almost exclusively on predictive modeling, outbreak forecasting, and the development of early warning systems (Santangelo et al., 2023). This concentration of research efforts has relegated advanced exploratory analysis to a secondary role, resulting in limited attention to the integration of dimensionality reduction techniques with unsupervised clustering algorithms for the analysis of national surveillance repositories on neglected tropical diseases.

This methodological gap represents a critical limitation in the current literature, as it restricts the identification of complex epidemiological patterns and latent interactions that traditional predictive approaches are not designed to capture (Hristamyan, 2025; Keshavamurthy et al., 2022).

### 2.4 Research gap and contribution

There are epidemiological studies based on traditional statistical and descriptive analysis methods that highlight research gaps regarding Carrion's disease. These studies propose the use of machine learning approaches to uncover hidden patterns, identify subgroups with specific risk profiles, and provide evidence to support the development of more targeted surveillance and intervention strategies.

The review of the scientific literature reveals a gap in the analysis of this disease: no previous studies have evaluated unsupervised machine learning algorithms using this institutional repository or databases with equivalent structures within the Peruvian context. Recent epidemiological studies have been limited to mapping the temporal and geographic distribution of Carrion's disease in the country, while their methodological scope remains constrained to conventional statistical tools, cross-tabulations, and purely descriptive segmentation approaches (Vera-Mendoza, 2025).

This study addresses this methodological gap. We propose an integrated approach that evaluates and compares complementary dimensionality reduction techniques—including Principal Component Analysis (PCA), Multiple Correspondence Analysis (MCA), and Factor Analysis of Mixed Data (FAMD)—together with consistent clustering algorithms (K-Means and BIRCH), applied on a large scale to the national epidemiological surveillance records of Carrion's disease in Peru during the 2000–2024 period.

Although these techniques belong to the standard toolkit of machine learning, integrating them into a single workflow makes it possible to reproduce and systematically characterize previously described epidemiological profiles, providing a more transparent and automated methodological approach for analyzing the temporal, geographic, and clinical distribution of disease cases. This analytical framework goes beyond the traditional descriptive interpretations and linear tabulations that commonly dominate official reports (Wang et al., 2019; Lu and Uddin, 2023).

Under this premise, the core scientific contribution lies not only in the statistical processing itself but also in the architecture of the methodological workflow. We designed a reproducible framework for the analysis of neglected tropical diseases that directly supports three critical public health objectives: epidemiological stratification in highly endemic areas, the technical auditing of the quality and consistency of health records, and the generation of quantitative evidence to optimize decision-making and resource allocation within the healthcare sector (Al Meslamani et al., 2024).

## 3 Materials and methodology

### 3.1 Description of the dataset

The dataset used in this study was obtained from the Peruvian Government's Open Data Platform, specifically from the cases reported by the Centro Nacional de Epidemiología, Prevención y Control de Enfermedades (CDC PERU) through the Red Nacional de Epidemiología (RENACE) (Plataforma Nacional de Datos Abiertos, 2026).

This public health surveillance system in Peru contains a dataset comprising more than 45,000 records spanning the period from 2000 to 2024, including geographic information (departments, provinces, and districts), demographic data (age and sex), dates, and the clinical classification of Carrion's disease.

As an open-access repository, the dataset underwent a prior strict anonymization process; consequently, the data matrix contains no names, personal identification records, addresses, or any other variables that could serve as direct or indirect patient identifiers.

Since the methodological design did not require any intervention involving or direct contact with human subjects, nor did it involve the processing of de-anonymized confidential information, the study protocol was exempt from the requirement to obtain informed consent or to undergo review by an institutional bioethics committee.

This exemption is consistent with international standards and local legislation governing the scientific use of indexed public records. Consequently, the study was conducted within an ethical framework, ensuring the principles of confidentiality, integrity in metadata management, and compliance with the current guidelines for the reuse of open data promoted by the Peruvian Government.

The data dictionary corresponding to this dataset is presented in Table 1, where a detailed description of each variable, as well as the data source, is provided.

**Table 1:** Data dictionary of the epidemiological surveillance dataset (2000–2024).

Variable	Data type	Description	Domain
departamento	Categorical	Administrative department where the case was reported.	Text labels (19 distinct values)
provincia	Text	Province where the case was reported.	Text labels
distrito	Text	District where the case was reported.	Text labels
localidad	Text	Locality where the reported case occurred.	Text labels
enfermedad	Categorical	Clinical classification of Carrion's disease recorded in the surveillance system.	Text labels (2 distinct values)
ano	Real number	Calendar year of case notification.	Numeric values
semana	Real number	Epidemiological week of case notification.	Numeric values
diagnostic	Categorical	Diagnostic code assigned to the reported case.	Alphanumeric codes (2 distinct values)
diresa	Real number	Identifier of the Regional Health Directorate responsible for reporting the case.	Numeric codes
ubigeo	Real number	Geographic location code associated with the reported case.	Numeric geographic codes
localcod	Real number	Locality identification code used by the surveillance system.	Numeric locality codes
edad	Real number	Age of the reported patient.	Numeric values
tipo_edad	Categorical	Unit used to record the patient's age.	Text labels (3 distinct values)

### 3.2 Data preprocessing

The retained features, their data types, domains, and the number of categories or value ranges used in the final analysis are presented in Table 1. To incorporate the categorical variables into the dimensionality reduction process, detailed in Section 3.3, appropriate encoding strategies were applied before the subsequent clustering algorithms. As a starting point for the data analysis, a data cleaning process was conducted, during which 669 duplicate records, representing 1.45% of the total dataset, were identified and removed through a complete row-by-row comparison across all variables.

Outlier detection was performed using Tukey's rule (1.5 × IQR) applied to the normalized edad variable. The calculated values were Q1 = 5 years, Q3 = 25 years, and IQR = 20 years, resulting in a valid interval ranging from 0 to 55 years (Tukey, 1977). Observations falling outside these limits were considered outliers and excluded from the analysis. In total, 1,917 records (4.22% of the dataset) were removed, resulting in a final analytical sample of 43,534 cases.

Once these anomalies had been identified, their clinical and statistical impact on the overall architecture of the repository was evaluated. Through selective pruning, only the records that threatened the convergence of the algorithms were excluded, while those reflecting the true variability of the phenomenon were retained. This methodological decision improved the stability of the preprocessing stage without compromising the representativeness of the subsequent modeling.

**Figure 1:**
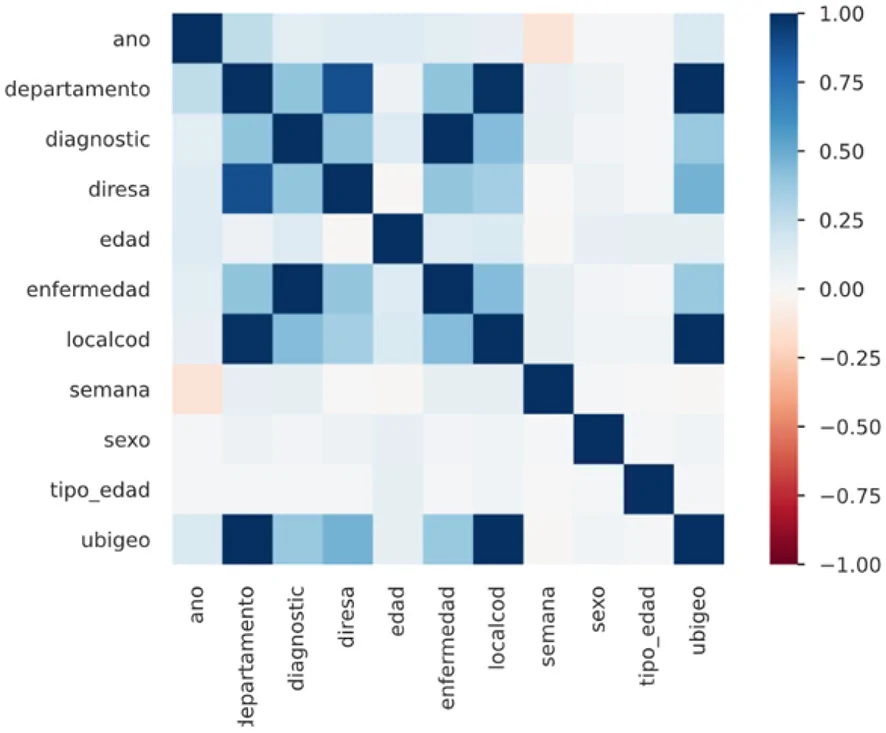
Pearson correlation matrix among numerical variables of the dataset.

Subsequently, the Pearson correlation coefficient was applied to the numerical variables (Figure 1), whereas Cramér's V was used to evaluate the categorical variables. The numerical variables showed no significant multicollinearity (|*r*| < 0.7), indicating independence among the quantitative features. However, the categorical variables exhibited substantial redundancy, particularly among the geographic and diagnostic attributes.

The geographic variables (departamento, provincia, distrito, and ubigeo) exhibited an almost perfect correlation according to Cramér's V, as presented in Table 2. Since ubigeo contains equivalent geographic information, the variables departamento, provincia, and distrito were considered redundant.

**Table 2:** Redundant variables eliminated by association analysis (Cramér's V).

Removed variable	Retained variable	Justification
Provincia	Departamento	Hierarchical geographic redundancy
Distrito	Departamento	Hierarchical geographic redundancy
Ubigeo	Departamento	Encodes the same geographic hierarchy
Diresa	Departamento	Highly correlated geographic information
Enfermedad	Diagnostic	Diagnostic code provides a standardized clinical representation

Similarly, the variable diresa was removed because of its high correlation with ubigeo (*V* = 0.92). This correlation-based feature selection reduced the number of variables to six while preserving their informational content.

The systematic audit of the repository's integrity determined that missing records were concentrated almost exclusively in the fine-grained georeferencing fields. The variable localcod contained 78.58% missing records, whereas localidad exhibited only 0.52% missing values. Apart from these two variables, the set of variables considered critical for the epidemiological modeling process showed 100% completeness.

Following the removal of duplicate records, missing data were handled using a complete-case analysis approach. This selective filtering excluded only those records containing inconsistencies within the essential demographic variables, defined by departamento, sexo, edad, and the age measurement unit (tipo_edad).

After applying this filtering procedure, the proportion of excluded records remained well below the 5% threshold, representing a marginal proportion that the literature considers acceptable for minimizing the risk of selection bias and preserving the statistical representativeness of the sample (Little and Rubin, 2019). The final stage of data cleaning focused on the age variable. To standardize the data for machine learning, a normalization procedure was developed by jointly processing the edad and tipo_edad variables, converting and harmonizing all observations originally recorded in months or days into a single metric expressed in years.

One-Hot Encoding was applied to variables with a large number of categories; however, this introduced a high-dimensionality problem. To mitigate this issue and obtain more compact representations, dimensionality reduction techniques appropriate for each data type were applied: PCA for numerical variables, MCA for categorical variables, and FAMD for mixed data. Subsequently, the K-Means algorithm was applied to evaluate clustering performance across all generated representations (KMeans, 2025).

The entire experimental workflow was conducted on the cloud-based Google Colaboratory (Google Colab) platform, with all computations performed exclusively using CPU resources (Aron and Estefani, 2026). The workflow was implemented in Python 3.12.13, with data processing and machine learning algorithms developed using a software ecosystem fixed to specific library versions: Pandas 2.2.2 and NumPy 2.0.2 for data engineering, Scikit-learn 1.6.1 together with SciPy 1.16.3 for modeling and scientific computing, and Prince 0.19.0 as the specialized library for dimensionality reduction on mixed data (Pedregosa et al., 2011).

### 3.3 Determination of the optimal number of dimensions

Following the analysis described in the previous section, the variables presented in Table 3 were used to proceed with the dimensionality reduction stage—specifically through FAMD or MCA. This process was based on evaluating the intrinsic clusterability of the projected space. This approach is founded on a clear methodological principle: for dimensionality reduction to be useful in clustering tasks, its underlying structure must preserve or enhance the separation between the latent clusters (Assani-Amate et al., 2026).

**Table 3:** Final set of six variables retained for clustering.

Variable	Data type	Domain	Distinct values/range
departamento	Categorical	Geographic	19 distinct values
diagnostic	Categorical	Clinical	2 distinct values
sexo	Categorical	Demographic	2 distinct values
edad_anios	Numerical	Demographic	0–55 years
semana	Numerical	Temporal	1–53
ano	Numerical	Temporal	2000–2024

To operationalize this criterion, the K-Means algorithm was incorporated as an auxiliary tool by configuring a controlled evaluation scenario with *k* = 5 on the transformed data matrix.

The geometric structure of the reduced space was assessed using a set of internal validation metrics selected for their mathematical complementarity (Milligan and Cooper, 1985). The Silhouette coefficient was used to evaluate the balance between intra-cluster cohesion and inter-cluster separation; the Davies–Bouldin index was employed to minimize the ratio between within-cluster dispersion and cluster separation; and the Calinski–Harabasz index was used to favor topologies exhibiting high compactness and strong separation among clusters (Shi et al., 2021).

Because the dataset contains numerical variables (edad, ano, and semana) together with categorical variables (departamento, sexo, and diagnostic), the methodological design required the application of Factor Analysis of Mixed Data (FAMD), a mathematical framework specifically developed to jointly embed and encode both variable types within the same latent space (Pagès, 2014).

This technical choice was motivated by the inherent limitations of conventional techniques. On the one hand, Principal Component Analysis (PCA) is designed exclusively for continuous variables, where forcing the inclusion of categorical variables through One-Hot Encoding introduces geometric distortions and biased variance representations. On the other hand, Multiple Correspondence Analysis (MCA) is limited to qualitative variables, preventing the direct incorporation of numerical information (Abdi and Williams, 2010; Greenacre, 2017).

Likewise, although non-linear approaches such as t-SNE and UMAP are widely used for exploratory visualization in high-dimensional spaces, their objective functions are designed to preserve local relationships between observations. This local emphasis tends to distort the global topology of the data, substantially limiting both the interpretability of the resulting dimensions and the deterministic reproducibility of the subsequent clustering process (Van Der Maaten and Hinton, 2008; McInnes et al., 2018).

Based on this reduced and optimized representation, clustering was subsequently performed using the K-Means algorithm. This sequential workflow was justified by the observation that the latent space generated by FAMD exhibited high intra-cluster compactness together with clear separation among clusters. This geometric consistency was reflected in a Silhouette coefficient of 0.8438 and a Davies–Bouldin index reduced to 0.2010. The convergence of these statistical indicators quantitatively demonstrated that the distribution of observations within the low-dimensional space satisfactorily fulfills the assumptions of spherical cluster structure and geometric compactness required for the proper operation of K-Means, thereby validating its use as the primary clustering algorithm and confirming that the resulting cluster boundaries are not artifacts produced by random noise within the repository (Rousseeuw, 1987; Davies and Bouldin, 1979).

The choice of K-Means over density-based (DBSCAN) or model-based (Gaussian Mixture) alternatives was deliberate and aligned with the geometric properties of the FAMD-projected space. Prior comparative work has shown that no single unsupervised algorithm is universally optimal, and that model selection should be driven by the structure of the data and the analytical goal (Lu and Uddin, 2023). Given that the FAMD projection yielded compact, convex, and well-separated point clouds—conditions under which centroid-based partitioning is most appropriate—K-Means was prioritized for its interpretability, deterministic reproducibility under a fixed seed, and scalability to the full national repository. Density-based methods, by contrast, are better suited to non-convex or noise-heavy structures not observed in the reduced space, while the exploratory aim of this study did not require the probabilistic soft-assignment of mixture models.

#### 3.3.1 PCA evaluation

For the numerical variables, the dataset included only three attributes; therefore, the PCA analysis retained 100% of the explained variance, making dimensionality reduction unnecessary. This is because these variables do not exhibit correlation, as explained in Section 3.2.

#### 3.3.2 MCA evaluation

For the categorical variables, the MCA method produced an appropriate factor representation. However, because MCA transforms each category into a separate column, the information is distributed across many low-dimensional components (Greenacre and Blasius, 2006). For this reason, different dimensionality configurations were evaluated using internal clustering validation metrics.

To determine the appropriate number of dimensions for MCA, the resulting values were comparatively analyzed using each of these metrics, as shown in Table 4, which presents the results of these evaluations.

**Table 4:** Dimensionality assessment with MCA.

Silhouette	Davies-Bouldin	Calinski-Harabasz	Dimensiones
**0.5395**	**0.6949**	**34,000.28**	2
0.4355	0.8219	18,799.14	3
0.4473	0.7543	15,224.25	4
0.4201	1.0785	9,831.22	5
0.3992	1.0504	4,758.64	10

Bold values indicate the best result for each evaluation metric.

Table 4 shows that the two-dimensional configuration is supported by the highest Silhouette coefficient (0.5395), the lowest Davies–Bouldin index (0.6949), and the highest Calinski–Harabasz index (34,000.28), indicating a more clearly defined cluster structure than the other evaluated configurations, with high intra-cluster cohesion and greater separation between clusters.

#### 3.3.3 FAMD evaluation

Because the dataset contains mixed variables, the FAMD method is appropriate for obtaining a joint representation that simultaneously preserves the variability of the numerical variables and the factor structure of the categorical variables. Similarly to the procedure followed for MCA, different dimensionality configurations were evaluated using internal clustering validation metrics, since cumulative explained variance is not a sufficient criterion in the presence of categorical variables encoded through indicator variables.

Table 5 shows that the two-dimensional configuration provides the most clearly defined cluster structure for the mixed data, achieving the optimal values for all three indices: the Silhouette coefficient (0.4716), the Davies–Bouldin index (0.5170), and the Calinski–Harabasz index (77,435.51).

**Table 5:** Dimensionality assessment with FAMD.

Silhouette	Davies-Bouldin	Calinski-Harabasz	Dimensiones
**0.4716**	**0.5170**	**77,435.51**	2
0.3692	0.7968	33,373.62	3
0.3859	0.7887	22,783.24	4
0.3194	0.9136	14,934.65	5
0.1620	1.5708	5,255.98	10

Bold values indicate the best result for each evaluation metric.

Therefore, the two-dimensional representation is considered the most appropriate for the subsequent clustering analysis, prioritizing the stability of the cluster structure and the interpretability of the results.

### 3.4 Determination of the optimal number of clusters

This process aimed to ensure the stability and interpretability of the clustering solution. To this end, a range of k values from k = 2 to k = 10 was evaluated using the internal validation metrics previously defined: the Silhouette coefficient, the Davies–Bouldin index, and the Calinski–Harabasz index, employing at this stage the optimal number of dimensions determined in the previous section.

Reproducibility during the clustering stage was ensured by controlling the hyperparameters of each algorithm. For the K-Means implementation, the process was configured using the optimized k-means++ initialization method, with 10 independent runs (n_init = 10) and a fixed random seed (random_state = 42) to eliminate variability between executions.

In contrast, the BIRCH algorithm was configured with a fixed cutoff radius (*threshold* = *0.5*) to control density during the construction of the Clustering Feature (CF) tree, following the original design of the algorithm (Zhang et al., 1996).

After this initial condensation stage, the algorithm performed a secondary clustering phase by systematically evaluating different values of the parameter *k*. This strategy provided a balanced comparison of the performance of both algorithms under a homogeneous computational environment.

Any other configuration parameters outside this controlled setting were left at the default values specified by the Scikit-learn library (Pedregosa et al., 2011).

#### 3.4.1 Application of K-Means with MCA

The K-Means algorithm was applied to the reduced dataset by combining the two-dimensional factor representation obtained through MCA for the categorical variables with the original numerical variables, which were previously standardized using the z-score technique through StandardScaler from the Scikit-learn library.

The analysis presented in Table 6 shows that the solution with *k* = 4 clusters provides the best overall performance for this dataset. This value of *k* is supported by both the Silhouette coefficient (0.2745) and the Calinski–Harabasz index (14,409.23), while also minimizing the Davies–Bouldin index (1.2239).

**Table 6:** Optimal number of clusters (k) with MCA + K-Means.

Silhouette	Davies-Bouldin	Calinski-Harabasz	*k*
**0.2745**	1.7666	11,168.80	2
0.2372	1.4526	12,292.69	3
0.2582	**1.2239**	**14,409.23**	4
0.2406	1.3047	12,712.91	5
0.2151	1.3899	11,826.08	6
0.1970	1.4815	11,038.99	7
0.1990	1.5955	10,321.31	8
0.1906	1.5339	9,664.72	9
0.1913	1.4799	9,068.64	10

Bold values indicate the best result for each evaluation metric.

Therefore, *k* = 4 was selected as the optimal number of clusters for K-Means using MCA and standardized numerical variables.

#### 3.4.2 Application of K-Means with FAMD

Similarly, the K-Means algorithm was applied to the two-dimensional factor representation obtained through FAMD for the mixed dataset. The results presented in Table 7 indicate that the configuration with *k* = *2* clusters is the most appropriate, as it achieves the highest Silhouette coefficient (0.8438) and the lowest Davies–Bouldin index (0.2010).

**Table 7:** Optimal number of clusters with FAMD + K-Means.

Silhouette	Davies-Bouldin	Calinski-Harabasz	*k*
**0.8438**	**0.2010**	25,043.80	2
0.5152	0.6329	53,436.48	3
0.5248	0.4792	55,115.57	4
0.4716	0.5170	77,435.51	5
0.4494	0.5520	90,937.24	6
0.4044	0.6253	94,582.11	7
0.3845	0.6713	**94,846.67**	8
0.3599	0.7385	92,184.83	9
0.3573	0.7555	89,409.56	10

Bold values indicate the best result for each evaluation metric.

Although the Calinski–Harabasz index presents a more favorable value at *k* = *8* (94,846.67), the selection of *k* = *2* is based on the agreement between the Silhouette and Davies–Bouldin metrics, prioritizing structural robustness and greater interpretability of the results.

Consequently, for the data reduced using FAMD, *k* = *2* was selected as the optimal number of clusters.

#### 3.4.3 Application of BIRCH with FAMD

Finally, the BIRCH algorithm was applied to the mixed data reduced through FAMD and subsequently standardized, with the aim of evaluating whether a more scalable clustering approach (BIRCH) would confirm the optimal solution previously obtained.

As in the case of K-Means with FAMD, the Calinski–Harabasz index favors a different partition, reaching its maximum value at *k* = 7 (41,065.14). However, the superiority of *k* = 2 in the other metrics, together with the previously obtained results, demonstrates that this bipartite structure is the most stable and best defined (Table 8). Overall, the evaluation of the optimal number of clusters shows that the results depend on the data representation used. While the combination of MCA with numerical variables suggests *k* = 4, the two approaches applied to the FAMD representation—both K-Means and BIRCH combined with K-Means—consistently converge to *k* = 2 as the most appropriate solution.

**Table 8:** Optimal number of clusters with FAMD + BIRCH.

Silhouette	Davies-Bouldin	Calinski-Harabasz	*k*
**0.8952**	**0.1556**	215.61	2
0.7753	0.5430	13,721.62	3
0.6841	0.3533	12,713.66	4
0.6827	0.3317	9,574.15	5
0.4453	0.4214	23,867.35	6
0.4331	0.4361	**41,065.14**	7
0.4298	0.5368	35,454.02	8
0.4298	0.4631	31,030.72	9
0.4105	0.5413	28,349.70	10

Bold values indicate the best result for each evaluation metric.

#### 3.4.4 Stability and sensitivity analysis of the model

To evaluate the robustness of the clustering solution, a stability analysis was conducted through multiple independent runs of the K-Means algorithm using the same methodological configuration.

The resulting Silhouette coefficients showed low variability and consistent behavior across iterations, indicating that the identified solution is stable with respect to different initializations (Hennig, 2006; von Luxburg, 2010).

Additionally, a sensitivity analysis was performed by completely excluding the diagnostic variable (ICD-10 codes). The observed decrease in the Silhouette coefficient was minimal (Δ = 0.0385), demonstrating that the cluster structure does not depend exclusively on this variable but is also supported by geographic, temporal, and demographic features. Overall, these results confirm the robustness of the proposed solution.

Collectively, the systematic comparison of the three evaluated approaches—K-Means with MCA, K-Means with FAMD, and BIRCH with standardized FAMD—consolidates the selection of *k* = 2 as the most consistent solution for the mixed dataset.

## 4 Results

### 4.1 Best model (FAMD with K-Means)

In terms of model performance, the combination of FAMD and K-Means consolidated the most suitable and consistent architecture for clustering the dataset. This quantitative superiority was directly reflected in the highest Silhouette coefficient (0.8438), together with the most pronounced reduction in the Davies–Bouldin index (0.2010), a pattern that indicates the formation of clusters that are clearly compact and well separated from each other.

The underlying efficiency is attributed to the intrinsic capability of FAMD to simultaneously encode numerical and categorical variables within a low-dimensional Euclidean space (Ufeli et al., 2025). This process mitigates peripheral statistical noise and preserves the latent structural relationships of the original matrix, providing an optimized projection that the K-Means algorithm exploits to isolate highly homogeneous groupings.

Consequently, this methodological coupling demonstrated strong sensitivity in capturing the marked heterogeneity present in the national surveillance records, validating it as the most robust and appropriate analytical approach for the objectives of this study.

#### 4.1.1 On the magnitude and robustness of the silhouette coefficient

The Silhouette coefficient obtained for the FAMD + K-Means solution (0.8438) is relatively high, higher than values typically reported in complex epidemiological clustering studies. This magnitude is largely explained by the dominance of a single categorical axis, geographic location (Department), which showed substantially stronger association with cluster membership (Cramér's *V* = 0.6119), while sex and diagnosis contributed negligibly (*V* = 0.0088 and 0.0244, respectively).

When a single categorical feature with an uneven category distribution—such as the near-exclusive concentration of Cluster 1 in Lima— plays the largest role in the factorial structure, FAMD tends to project the associated observations into a more separated region of the latent space, a well-recognized geometric property of factorial methods applied to imbalanced categorical data (Pagès, 2014; Greenacre, 2017) that can increase apparent separation.

Importantly, this separation cannot be solely attributed to the diagnostic coding: excluding the ICD-10 variable altered the Silhouette coefficient only marginally (Δ = 0.0385, Section 3.4.4), the clustering solution remained stable when additional FAMD dimensions were retained, and the bootstrap analysis showed low dispersion of the Silhouette coefficient together with stable centroid positions across resampled datasets. Taken together, these findings indicate that the high Silhouette coefficient reflects a consistent and robust geographically driven partition of the surveillance data. Although the factorial projection may accentuate the numerical separation between groups, it does not appear to generate the clustering structure itself; the observed separation should therefore not be overinterpreted as evidence of distinct clinical or biological subtypes, but rather as a geographically and temporally driven epidemiological pattern.

It should be noted that the mean Silhouette obtained during bootstrap resampling (=0.49) is not directly comparable to the coefficient of the final solution (0.8438, Table 7): the former quantifies the stability of the cluster structure across perturbed subsamples, whereas the latter reflects the quality of the definitive partition fitted on the complete dataset. The lower resampled value is therefore expected and should be interpreted as evidence of structural robustness rather than as a discrepancy in clustering quality.

### 4.2 Statistical comparison between clusters

**Figure 2:**
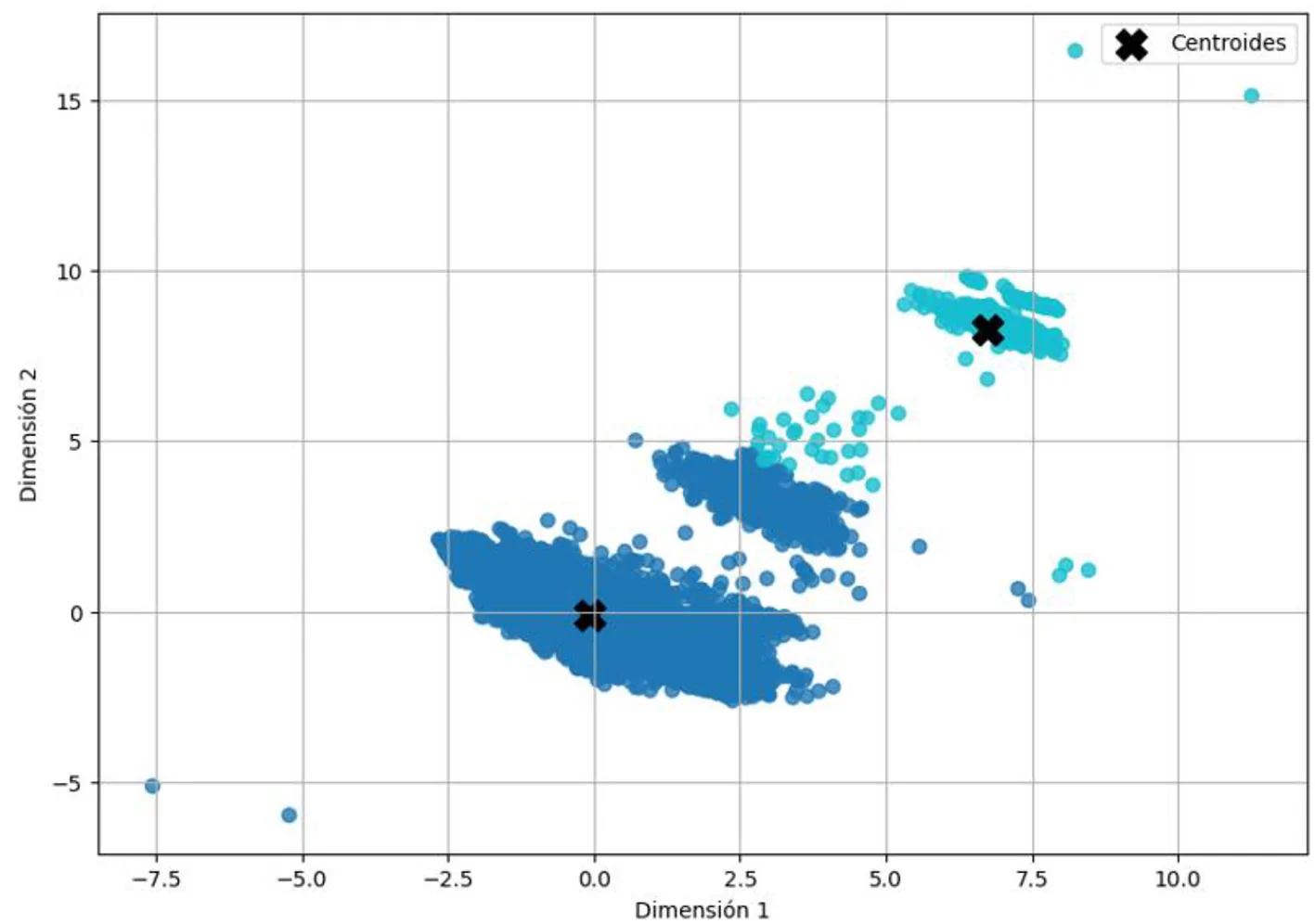
Distribution of the two clusters in the two-dimensional FAMD space.

**Figure 3:**
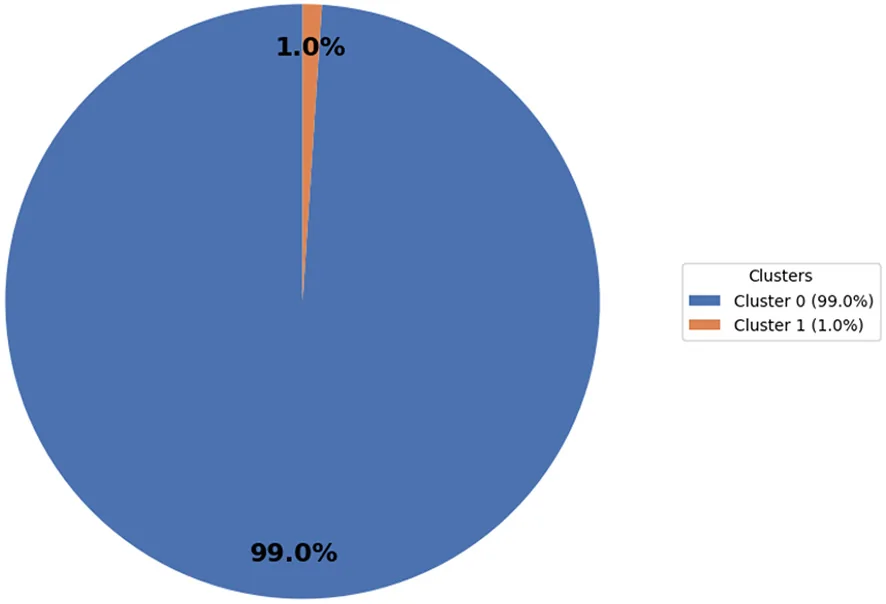
Two-dimensional projection of the records with FAMD and K-Means.

Before analyzing the statistical differences between both groups, the two-dimensional projection obtained through FAMD (Figure 2) and the spatial distribution of the clusters (Figure 3) show a clearly defined separation between the two groupings, evidencing that the solution obtained by K-Means presents a compact and well-differentiated structure.

The descriptive interpretation of the clusters incorporated inferential statistical tests aimed at verifying whether the observed profiles correspond to real underlying dynamics or merely to sampling fluctuations.

For the analysis of numerical variables—specifically age, epidemiological week, and year—the non-parametric Mann–Whitney U-test, shown in Table 9, was applied due to the non-normal nature of the distributions.

**Table 9:** Comparison of numerical variables between clusters (Mann–Whitney U-test).

Variable	Media C0	Media C1	U-stat	*p-value*
año	2005.482	2011.392	10,253,641.0	4.3386e-02
semana	24.855	21.675	10,913,086.0	7.7394e-06
edad_anos	15.148	19.239	7,986,065.5	5.4919e-11

For categorical variables (sex, diagnosis, and department), the comparison was conducted using the Chi-square (χ^2^) test, presented in Table 10, complemented with Cramér's *V* coefficient to assess the true magnitude of the effect size (McHugh, 2013; Tomczak and Tomczak, 2014; Cohen, 2013).

**Table 10:** Comparison of categorical variables between clusters (χ^2^ and Cramér's V).

Variable	Chi^2^	*p-value*	V de Cramér
departamento	16,297.68	0.0000e+00	0.6119
sexo	4.34	3.7129e-02	0.0088
diagnostic	26.79	2.2646e-07	0.0244

**Figure 4:**
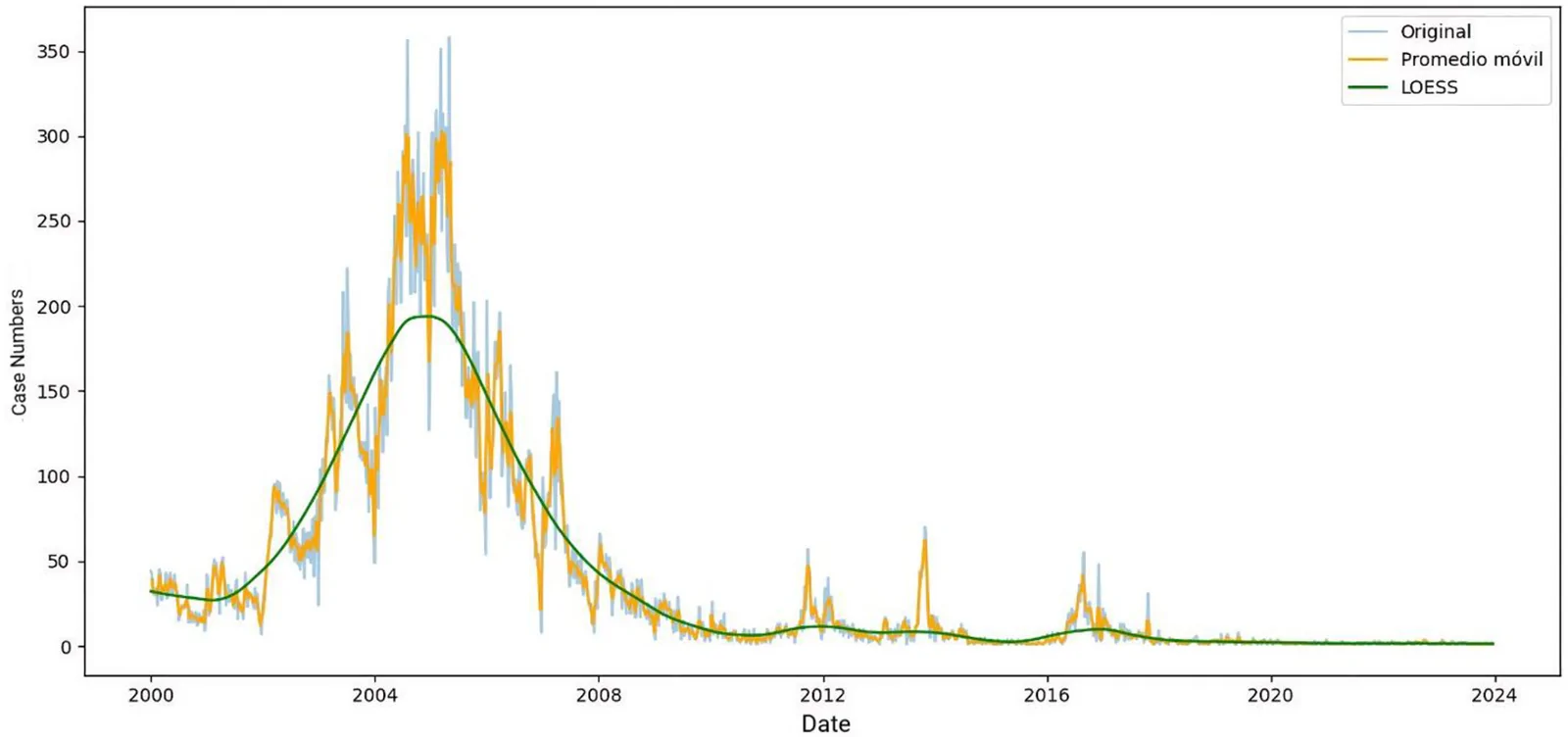
Number of reported cases per year.

**Figure 5:**
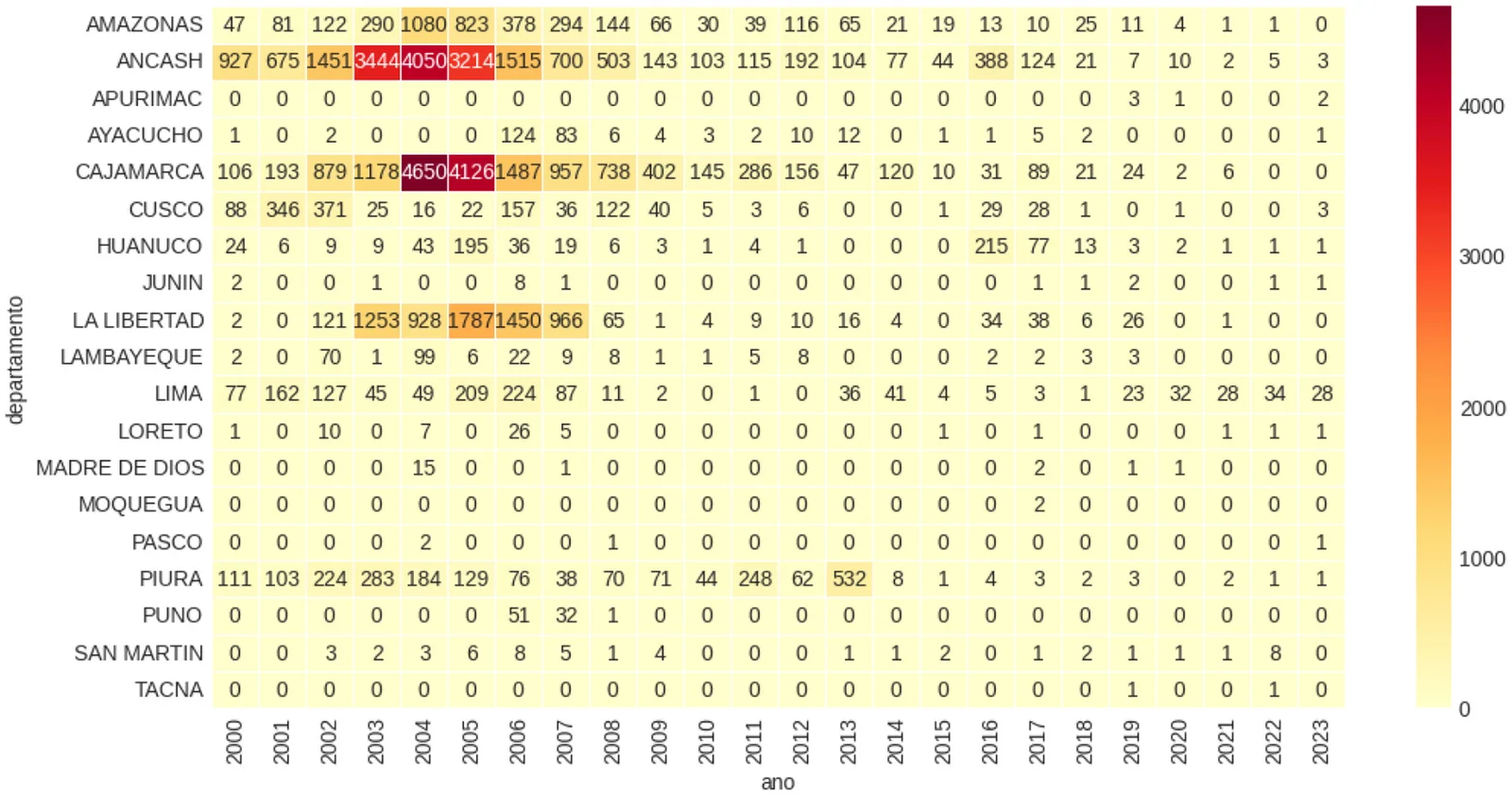
Departments with the highest number of cases per year.

**Figure 6:**
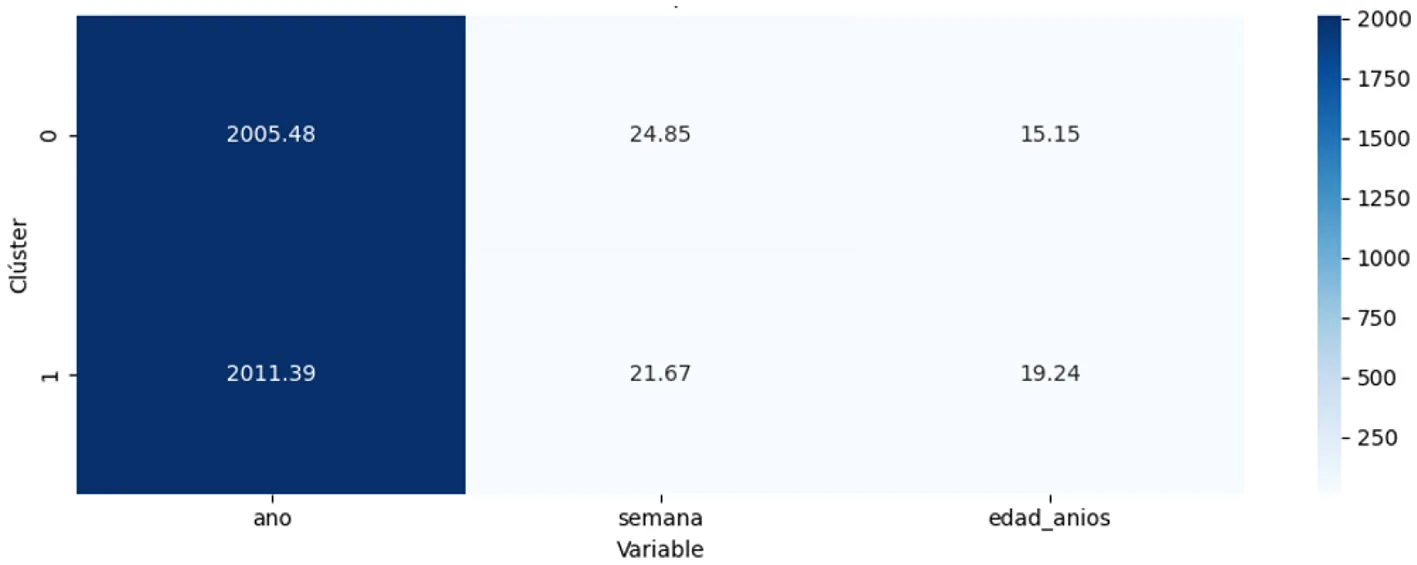
Comparación de variables numéricas por clúster.

The results supported statistically significant differences in the temporal distribution of cases (Figure 4), the geographic distribution (Figure 5), and age ranges (Figure 6). Among all variables, department exhibited the strongest association with the clustering structure, with a Cramér's *V* coefficient of 0.6119; this value indicates a strong effect size and positions geographic origin as the primary driver of segmentation.

In contrast, the behavior of the sex variable, although statistically significant due to the large sample size, showed a very small effect size (*V* = 0.0088). This indicates that, in practice, patient gender contributes minimally to distinguishing clinical structures. These quantitative contrasts serve as an important validation check, demonstrating that the extracted clusters represent tangible epidemiological profiles rather than random deviations from the surveillance repository.

#### 4.2.1 Cluster 0: majority group

Representing 99% of the analyzed observations, Cluster 0 is the dominant epidemiological profile within the national surveillance repository. This group, relative to Cluster 1, is supported by the validation metrics presented in Table 7. This geometric consistency aligns in near-perfect symmetry with the two-dimensional projection derived from the FAMD analysis (Figures 2, 3), where Cluster 0 is organized as a dense, compact point cloud, clearly separated from the second partition, showing a distinct separation between both groups.

The inferential statistical tests detailed in Tables 9, 10 allow the identification of the variables that explain the separation between clusters. For numerical variables, Cluster 0 presents an average notification year of 2005.48, an average epidemiological week of 24.86, and a mean age of 15.15 years. These indicators show statistically significant differences compared to Cluster 1, with the Mann–Whitney U-test yielding *p* < 0.05 in all three cases. In contrast, for categorical variables, geographic origin emerged as the main driver of segmentation, with a Cramér's *V* coefficient = 0.6119, indicating a strong effect size.

The behavior of sex (*V* = 0.0088) and diagnosis type (*V* = 0.0244) reflects the opposite methodological pattern: although both variables achieved formal statistical significance due to the large sample size, their effect sizes were negligible. Overall, these quantitative contrasts demonstrate that the structure of Cluster 0 is primarily determined by geographic and temporal distribution of records, while patient gender and clinical manifestation play a secondary role with virtually no practical contribution to group differentiation.

**Figure 7:**
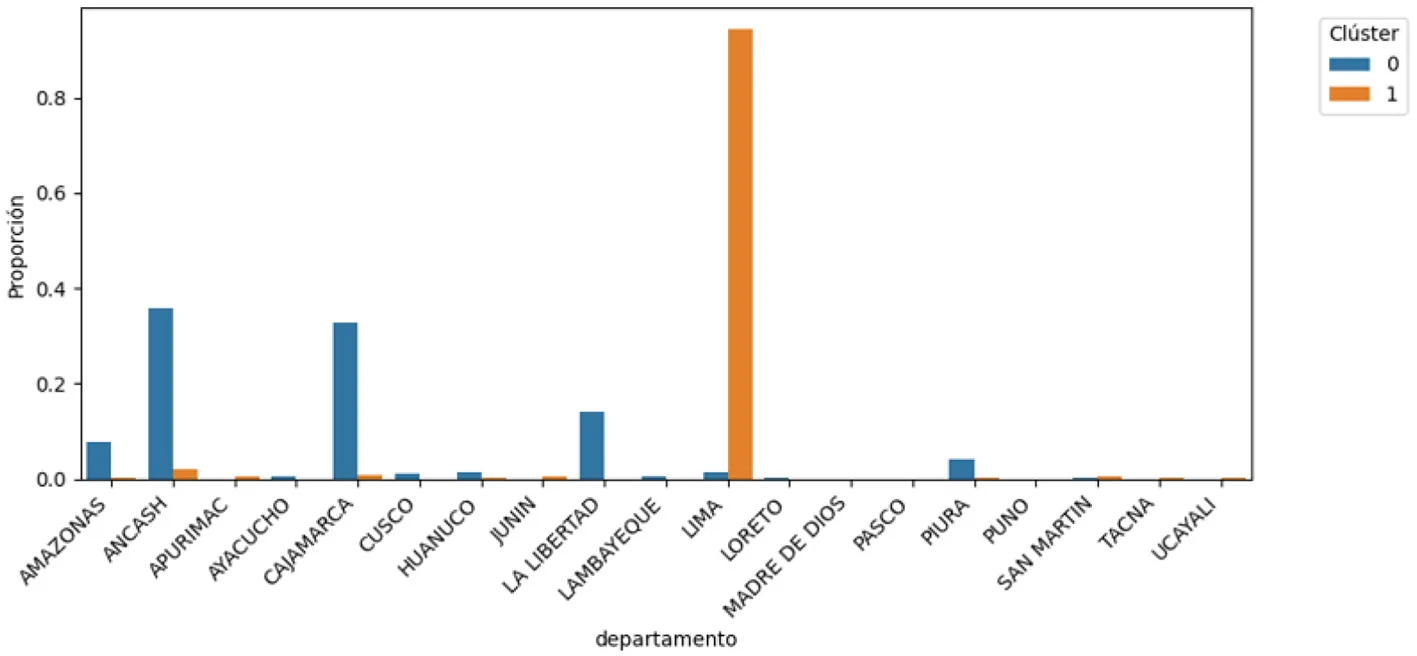
Distribution of department by cluster.

The geographic mapping in Figure 7 further clarifies this dynamic, showing that Cluster 0 concentrates the highest density of cases in the country's historically endemic regions, with Áncash (35.80%) and Cajamarca (32.79%) leading, followed by La Libertad (14.10%) and Amazonas (7.84%), a spatial distribution consistent with transmission hotspots reported by the Ministry of Health. The annual distribution of cases in the highest-incidence departments is shown in Figure 4.

**Figure 8:**
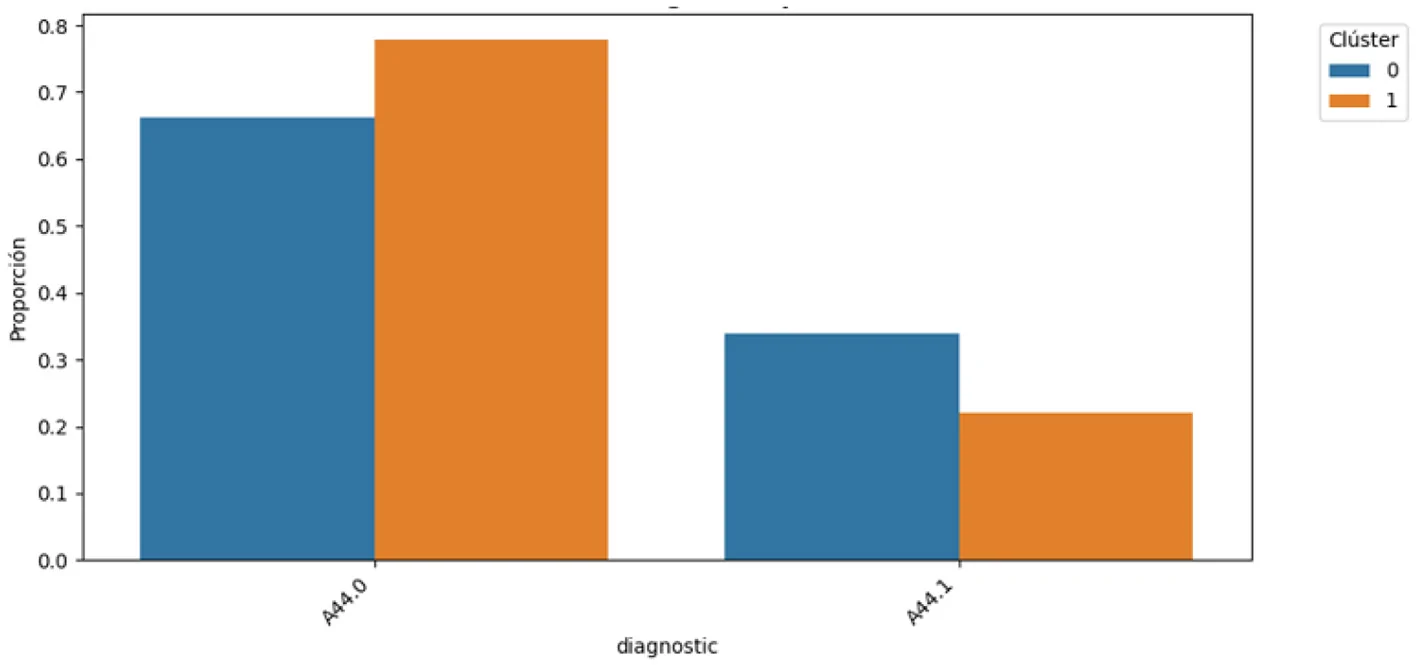
Distribution of ICD-10 diagnosis by cluster.

Regarding the clinical characterization in Figure 8, 66.20% of this cluster corresponds to code A44.0 (acute phase or Oroya fever), while the remaining 33.80% corresponds to A44.1 (chronic eruptive phase or Peruvian wart). Since the cleaned institutional matrix contains no records under the generic label A44.9, these percentages describe only the internal composition of the cluster and not variables driving its geometric construction, a behavior consistent with the marginal effect size observed in hypothesis testing.

**Figure 9:**
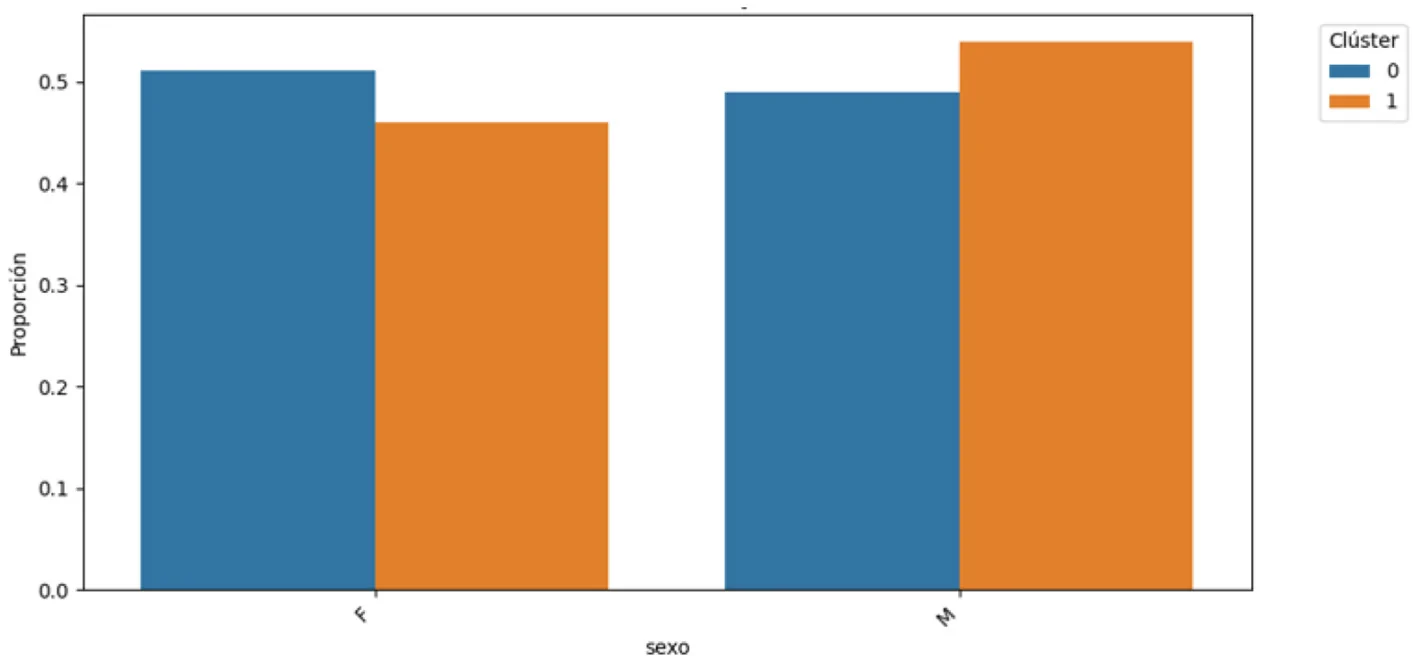
Distribution of sex by cluster.

Likewise, the gender distribution in Figure 9 shows a nearly balanced proportion between men and women, reinforcing the negligible role of this variable in the algorithmic segmentation.

In conclusion, Cluster 0 represents the basal and predominant epidemiological profile of Carrion's disease over the 2000–2024 period. Its clinical and demographic identity is defined by the high concentration of cases in traditional endemic areas, an earlier historical notification pattern, and a younger affected population compared to Cluster 1. The analysis confirms that the morphology of this large partition is explained almost exclusively by the geographic and temporal coordinates of the epidemiological phenomenon, while variations in clinical diagnosis and patient sex contribute minimally to the overall structure of the surveillance dataset.

#### 4.2.2 Cluster 1: minority group

Cluster 1 groups approximately 1% of the analyzed records, as shown in Figure 3, constituting the minority epidemiological profile identified in the dataset. Although this cluster represents a very small proportion of the sample, its clear separation in the FAMD space and the internal validation metrics indicate that it does not correspond to a residual group, but rather to a statistically well-defined profile. The spatial isolation of this group with respect to Cluster 0 is supported by the indicators in Table 7.

This separation is also observed in the two-dimensional projection obtained through FAMD (Figure 2), where Cluster 1 is located in a clearly distant region from the first partition; this behavior quantitatively demonstrates that the group forms a dense, cohesive, and well-defined cluster.

The inferential statistical tests detailed in Tables 9, 10 serve as an important analytical step to identify the variables governing this behavior. For numerical variables, Cluster 1 presents an average notification year of 2011.39—noticeably higher than the 2005.48 average of Cluster 0—a mean epidemiological week of 21.68, and a mean age of 19.24 years.

This set of indicators shows statistically significant discrepancies compared to the majority profile, with the Mann–Whitney U-test yielding *p* < 0.05 in all three cases; this result indicates that the surveillance system plays a key role in the differentiation between both groups. In contrast, for categorical variables, geographic location emerged as the dominant axis of segmentation, with a Cramér's *V* coefficient = 0.6119, indicating a strong effect size. The behavior of sex (*V* = 0.0088) and diagnosis type (*V* = 0.0244) follows the opposite methodological pattern: although both variables reached formal statistical significance due to the large sample size, their effect sizes were negligible. Overall, these quantitative contrasts demonstrate that the structure is driven by spatiotemporal dynamics, while patient gender and clinical manifestation play a secondary role with virtually no practical influence on group differentiation.

The geographic mapping (Figure 7) further highlights this asymmetry, showing that Cluster 1 concentrates 94.25% of its records in the department of Lima, while traditional endemic regions such as Áncash (1.99%) and Cajamarca (0.88%) drop to marginal proportions. This distribution breaks the dominance of historically endemic regions observed in Cluster 0, consistent with the strong effect size obtained for the department variable. Regarding clinical characterization (Figure 8), 77.88% of this group corresponds to code A44.0 (acute phase), while the remaining 22.12% corresponds to A44.1 (eruptive phase). Since the cleaned institutional matrix contains no records under the generic label A44.9, these percentages describe only the internal composition of the cluster.

Although the prevalence of the acute phase is slightly higher than in Cluster 0, the marginal effect size observed in hypothesis testing confirms that diagnostic variation did not drive cluster formation; these results indicate that segmentation was primarily determined by geographic and temporal differences rather than clinical classification. Likewise, the gender distribution (Figure 9) shows a nearly balanced proportion between men and women across both partitions, reinforcing the negligible role of sex in dataset segmentation.

In conclusion, Cluster 1 can be interpreted as a minority or peripheral epidemiological profile of Carrion's disease. Its clinical and demographic identity is defined by more recent notification years, a significantly higher average age, and a strong concentration in the department of Lima, distinguishing it from the traditional endemic pattern. Consequently, this structure appears to reflect transitions in surveillance dynamics and coverage rather than a distinct clinical variant of the disease. This finding highlights the methodological need for future research to incorporate more complex variables related to diagnostic capacity, real access to healthcare services, and regional sensitivity of the notification system to clarify the underlying origin of this pattern.

### 4.3 Cluster stability analysis

**Figure 10:**
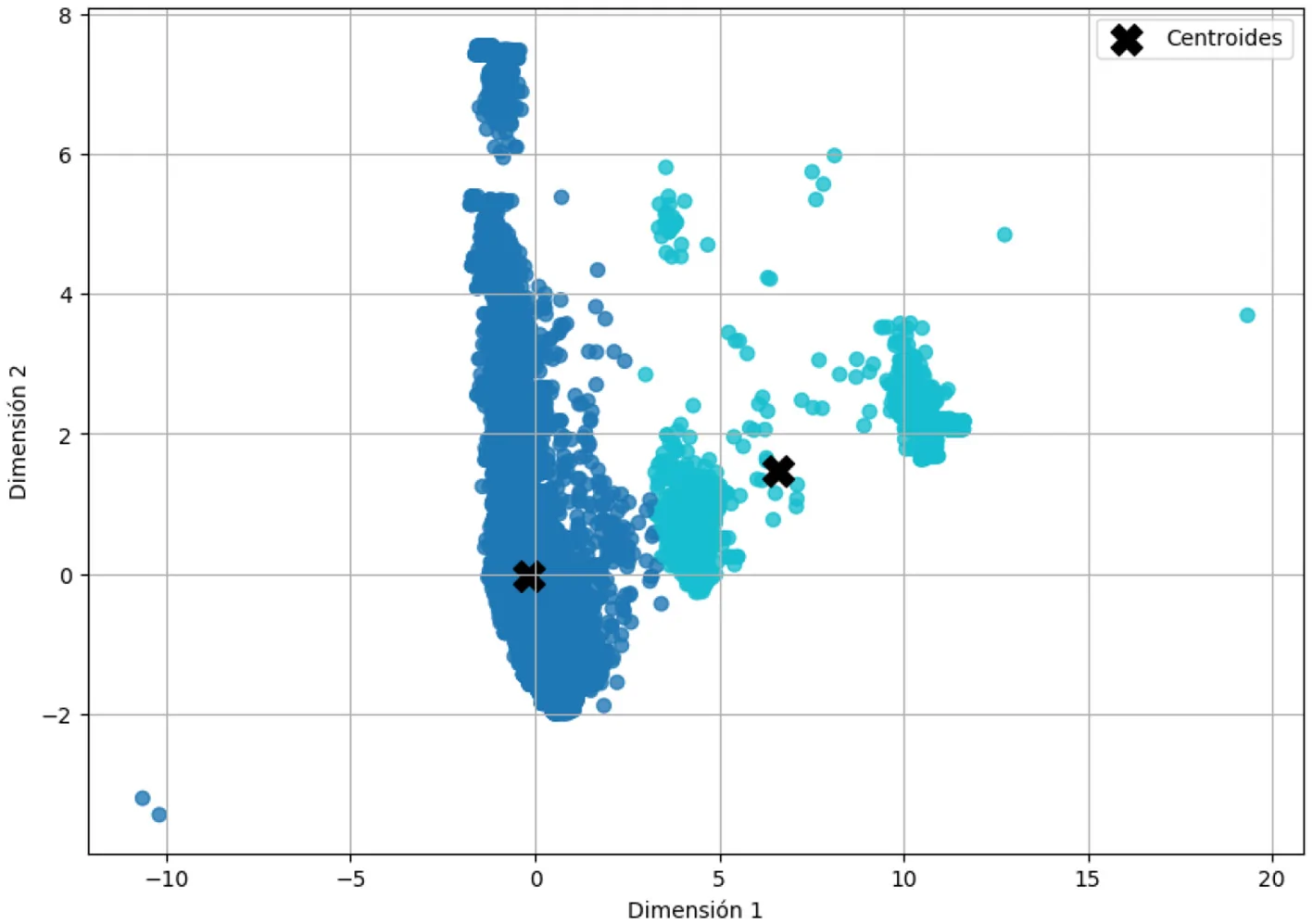
Distribution of clusters without the diagnosis variable (ICD-10).

The spatial projection of the clusters obtained after excluding the diagnostic variable is presented in Figure 10, where the persistence of the two-dimensional structure can be observed.

**Figure 11:**
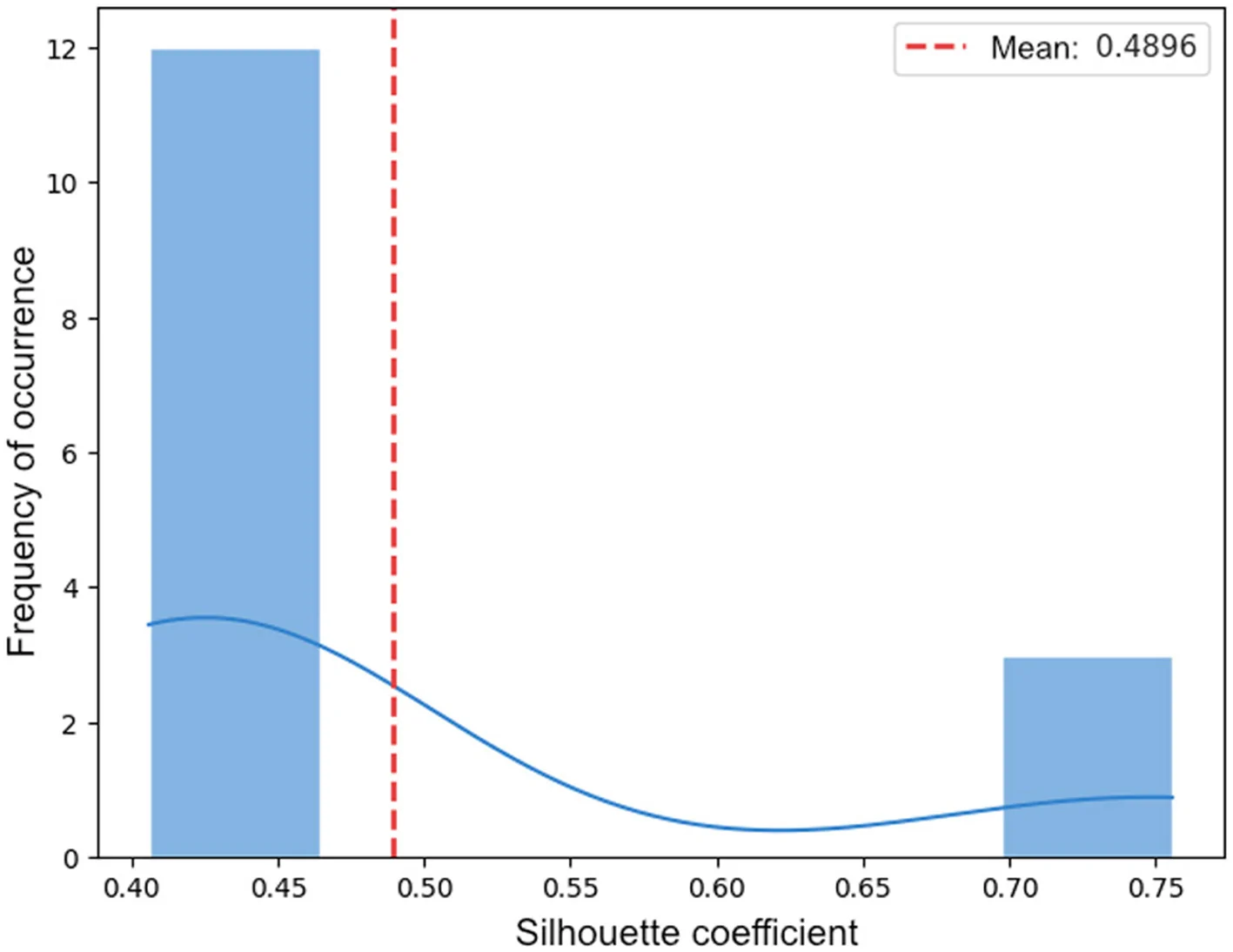
Density of the Silhouette coefficient in bootstrap.

**Figure 12:**
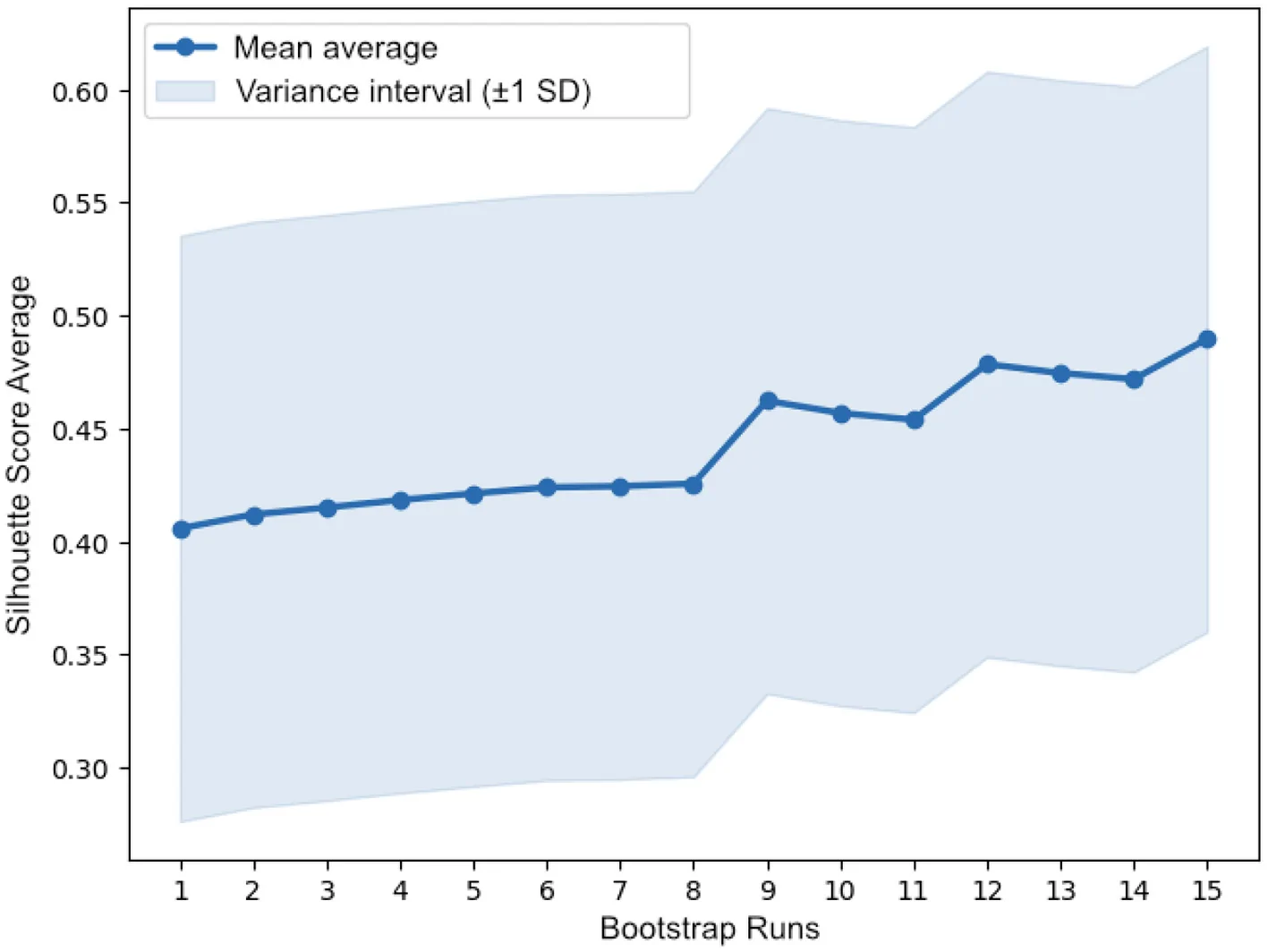
Statistical convergence of the Silhouette coefficient in bootstrap.

**Figure 13:**
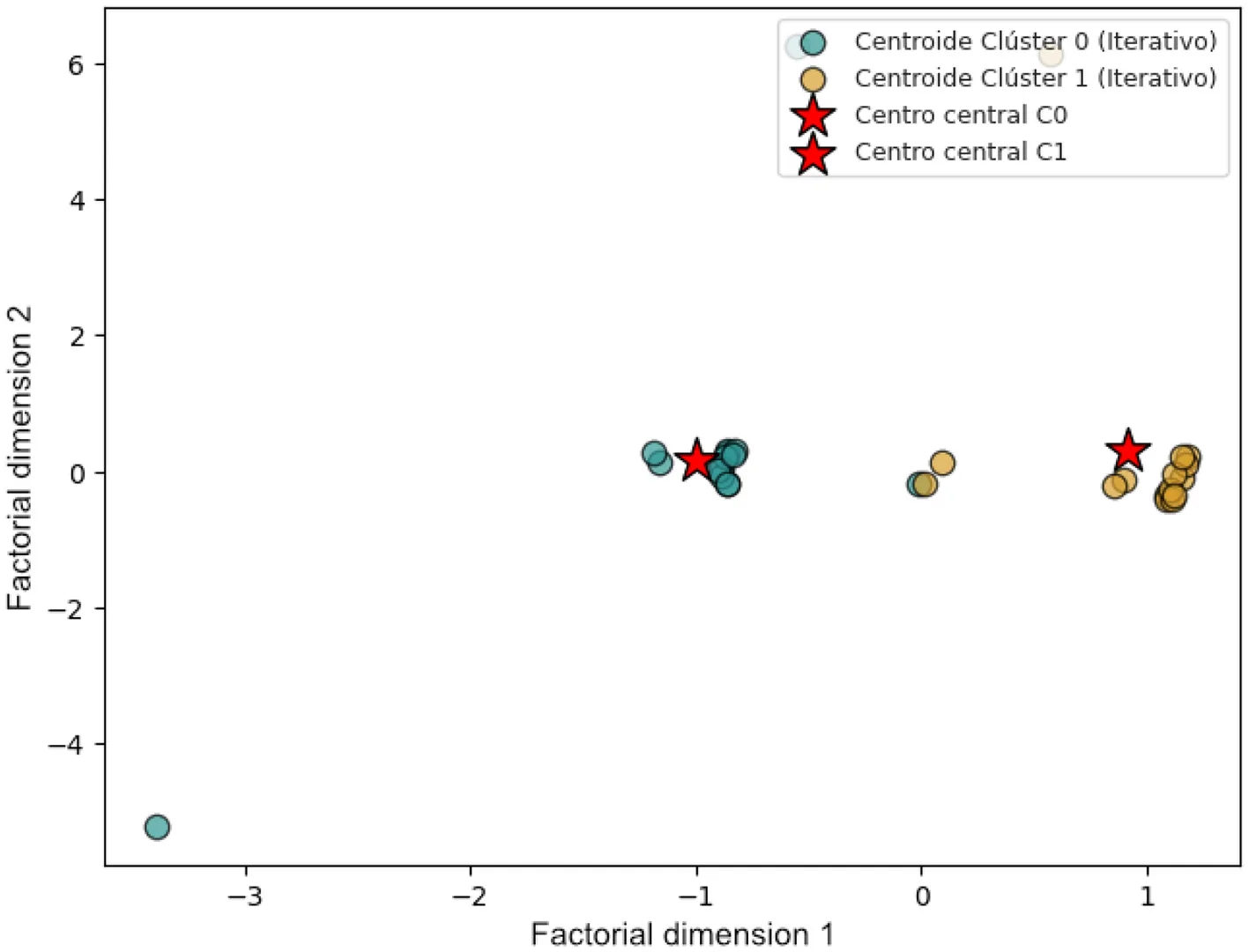
Stability of centroids against repository perturbations.

The consistency and robustness of the partitions were evaluated using a bootstrap procedure configured with 15 iterations. The bootstrap results showed that the dispersion of the Silhouette coefficients exhibited very low variability (Figure 11), with progressive convergence of the indicator throughout the resampling process (Figure 12). This structural stability was further supported by the mapping of the centroid spatial coordinates, whose geometric positions remained practically unchanged under perturbations introduced in the dataset (Figure 13).

Although the final clusters showed qualitative agreement with endemicity maps and transmission basins officially reported by the Ministry of Health (MINSA), this comparison represents an indirect and macro-level epidemiological approximation.

Consequently, the results should be interpreted as evidence of internal model stability rather than as definitive clinical or epidemiological validation, which would require independent studies and evaluation by public health specialists.

#### 4.3.1 Interpretation of the cluster size imbalance

The pronounced imbalance between Cluster 0 (99.0%) and Cluster 1 (1.0%) deserves further methodological examination, since extreme size asymmetries can, in principle, reflect either a underlying epidemiological subgroup or an artifact of the clustering procedure itself. Several observations are consistent with the former interpretation.

First, an outlier-driven explanation is not supported by the available evidence. Statistical outliers in the age variable were already identified and removed during preprocessing using Tukey's IQR rule (Section 3.2), prior to the clustering stage. A residual noise cluster would also be expected to show geometric instability, whereas Cluster 1 forms a dense, spatially cohesive group in the FAMD projection (Figure 2) whose centroid position remains stable across bootstrap resampling (Figure 13) and whose Silhouette coefficient shows low variance across iterations (Figures 11, 12).

Second, a purely random reporting-artifact explanation is inconsistent with the internal coherence of Cluster 1. If the minority group reflected stochastic noise in data capture, no systematic association with time, age, or geography would be expected. Instead, Cluster 1 is associated with a significantly later average notification year (2011.39 vs. 2005.48), an older mean age (19.24 vs. 15.15 years), and a 94.25% concentration in the department of Lima, all differences were statistically significant at *p* < 0.05 (Table 9) and supported by the largest effect size observed in the study (Cramér's *V* = 0.6119 for department, Table 10).

Overall, this pattern is more consistent with a distinct epidemiological context than by a distinct clinical form of the disease. Lima is not a historically endemic region for Carrion's disease, unlike Áncash, Cajamarca, La Libertad, and Amazonas, which dominate Cluster 0 (Figure 7). The concentration of more recent, older-age cases in Lima is compatible with imported or migration-related cases from endemic areas, with improvements in diagnostic capacity and case notification in the capital over the study period, or with a combination of both.

Under this interpretation, Cluster 1 is unlikely to represent random noise but a secondary, minority epidemiological signal, whose small size is consistent with the low incidence of the disease outside traditional endemic corridors rather than solely reflecting a limitation of the clustering procedure.

From an epidemiological standpoint, this pattern is directly relevant to Carrion's disease surveillance: the emergence of a distinct, more recent case profile concentrated in a non-endemic urban center (Lima) may signal either the geographic expansion of transmission beyond the historical Andean corridors or, more likely, the increasing detection of imported cases in a setting with greater diagnostic capacity. Either interpretation carries operational implications, as it suggests that surveillance protocols calibrated for classic endemic regions may not fully capture the evolving spatial dynamics of the disease.

Nevertheless, this interpretation should be considered as a data-based hypothesis, not as a confirmed etiological or clinical finding. As noted in Section 5, verifying whether this pattern reflects a consistent importation of cases, a differential access to medical care, or an evolving surveillance sensitivity in Lima would require additional complementary data on patient mobility, diagnostic infrastructure, and case investigation records, which are not available in the current institutional repository.

## 5 Study limitations

Although the proposed methodological approach for identifying epidemiological profiles using unsupervised machine learning is stable, the interpretation of the final findings must be qualified in light of several intrinsic limitations of the experimental design.

First, there is a dependency on the set of variables included, constrained by the feature space available in the institutional repository, since the clustering process was exclusively driven by the fields already present in the national surveillance database: department, sex, diagnosis, age, epidemiological week, and year. This technical constraint prevented the inclusion of additional clinical variables, environmental factors, socioeconomic records, or indicators related to real access to healthcare services—an external information ecosystem that would be critical to enrich cluster characterization in future studies. This data limitation is compounded by the inherently exploratory and non-causal nature of the analytical paradigm adopted.

The resulting clusters represent statistical configurations derived from similarities observed among records; therefore, they must not be interpreted as definitive clinical taxonomies or evidence of medical causality. Their value lies in serving as epidemiological hypotheses to guide future investigations rather than as confirmatory conclusions.

Furthermore, the configuration of the diagnostic variable introduces methodological constraints and a potential risk of classification bias that must be interpreted cautiously. The cleaned dataset distinguishes only two codes from the International Classification of Diseases (ICD-10): A44.0 for the acute phase and A44.1 for the eruptive phase, with no records under A44.9, which corresponds to unspecified bartonellosis as defined in the official Ministry of Health classification.

This lack of granularity limits the study's ability to assess the true quality of institutional coding or to track the prevalence of unspecified cases. This issue is reflected in the notably low effect size observed in cluster separation, with a Cramér's *V* coefficient = 0.0244 reported in Table 10.

This methodological ambiguity is also reflected in the evaluation of regional disparities in access to healthcare services, where the geographic differences observed between clusters in Table 10 may be driven more by unequal regional diagnostic capacity than by the underlying epidemiology of the disease itself. Due to the absence of external indicators on hospital coverage by region, this hypothesis remains open. Ultimately, the generalizability of the study is strictly limited to the analyzed dataset; therefore, extrapolation of this workflow to other countries, time periods, or healthcare surveillance systems should be performed with caution and validated through complementary studies.

Although cluster stability was assessed through a bootstrap procedure, showing low variability in the Silhouette coefficient and consistent centroid localization across resamples, this procedure represents only an internal validation strategy. Consequently, the identified profiles should be indicated through studies involving independent datasets and evaluation by epidemiology or public health experts, in order to verify their reproducibility and usefulness in contexts beyond the one analyzed.

Beyond the internal resampling procedures implemented in this study, further validation could strengthen confidence in the identified clustering structure through three complementary approaches. One possible strategy is replication using independent regional or international bartonellosis surveillance datasets, which would help assess would help assess the generalizability of the findings beyond the Peruvian surveillance system. Another complementary approach is independent evaluation by infectious disease specialists and public health epidemiologists, which could determine whether the identified profiles are clinically and operationally meaningful. A further line of validation would be prospective assessment using future surveillance data, which would help evaluate the temporal stability of the identified clusters under evolving epidemiological conditions. These validation strategies were beyond the scope of the present study and should therefore be considered when interpreting the external applicability of the reported clustering solution.

## 6 Conclusions

From an epidemiological perspective, this study reveals that the national surveillance records of Carrion's disease are organized around two structurally distinct profiles whose separation is governed primarily by geography and time rather than by clinical or demographic factors. The dominant profile reproduces the historically endemic Andean pattern (Áncash, Cajamarca, La Libertad, Amazonas), while a minority profile—more recent, older, and concentrated in non-endemic Lima—suggests an evolving spatial dynamic compatible with case importation or improved urban diagnostic capacity. Beyond the algorithmic result, this finding contributes to the understanding of the disease by showing, in a data-driven manner, that the epidemiology of Carrion's disease in the 2000–2024 period is not spatially static and that surveillance strategies may need to account for signals emerging outside the traditional transmission corridors.

In methodological terms, the results demonstrate that the integration of Factorial Analysis of Mixed Data (FAMD) with the K-Means algorithm constitutes an appropriate approach for exploring the internal structure of the national surveillance records of Carrion's disease through unsupervised learning. By contrasting different strategies of spatial compression, the two-dimensional projection derived from FAMD shows clinical interpretability and suitability for partitioning, a behavior strictly validated through the numerical indices of Silhouette, Davies–Bouldin, and Calinski–Harabasz (Table 11). Far from being an arbitrary choice, this configuration was established after testing multiple dimensionality scenarios, an optimization strategy that allowed balancing intra-cluster cohesion and inter-cluster separation within a single workflow.

**Table 11:** Summary of k selection; stable solution *k* = 2 for mixed data.

Algorithms and representation	*k*	Sil	DB	CH
K-Means (MCA + Numerical)	4	0.2745	1.2239	14,409.23
K-Means (FAMD)	2	0.8438	0.2010	94,846.67
BIRCH + K-Means (Standardized FAMD)	2	0.8952	0.1556	41,065.14

For each algorithm–representation combination, the reported k corresponds to the value selected as optimal based on the joint criterion of Silhouette and Davies–Bouldin agreement (Sections 3.4.1–3.4.3). The Calinski–Harabasz value shown reflects the best score observed across the full range of k tested (2–10), which in some cases occurs at a different k than the selected solution (e.g., k = 8 for FAMD+K-Means, k = 7 for FAMD+BIRCH), consistent with Tables 6–8.

These epidemiological profiles were further supported by inferential statistical analyses of the principal numerical and categorical variables. Significant differences were observed in case chronology, geographic origin, and age, whereas the contribution of the diagnostic variable remained limited. This inferential evidence reinforced that the identified clusters represent meaningful epidemiological profiles rather than random fluctuations or artifacts of the surveillance repository, providing a solid quantitative foundation for their interpretation.

This inferential contrast acted as a crucial mathematical validator: it not only reinforced the methodological rigor of the experimental design against noise and random variability but also provided a solid quantitative foundation for interpretation.

To assess whether the clustering structure was mainly determined by the diagnostic variable (ICD-10 codes), a control experiment was conducted by completely excluding the diagnostic variable from the modeling process. The results showed that the Silhouette coefficient decreased only from 0.8438 to 0.8054 (Δ = 0.0385), while maintaining a stable clustering structure. This indicates that the identified clusters do not depend exclusively on diagnostic classification, but also reflect consistent differences associated with geographic, temporal, and demographic variables. Consequently, the identified patterns represent epidemiological profiles that complement diagnostic coding information and provide additional evidence useful for epidemiological surveillance (Saab et al., 2025; Al Meslamani et al., 2024).

These findings can be contextualized against prior unsupervised analyses of health data. Whereas, Wang et al. (2019) found demographic variables such as age and sex to be central to latent subgroup formation, in the case of Carrion's disease sex proved epidemiologically negligible (Cramér's *V* = 0.0088), and segmentation was instead dominated by geographic origin—underscoring a disease-specific structure rather than a generic demographic one. Likewise, comparative studies of unsupervised clustering on clinical datasets report that no single algorithm dominates across contexts (Lu and Uddin, 2023), which is consistent with our observation that the FAMD + K-Means coupling outperformed the MCA-based configuration for this particular mixed-type surveillance data. This contrast highlights the originality of applying a mixed-data factorial approach to a neglected tropical disease repository, a combination not previously reported in the Peruvian context.

From a technical standpoint, the methodological refinements and improvements introduced during the technical review phase significantly strengthened the transparency and reproducibility standard of the study. The workflow was reinforced through a strict control protocol that included full computational environment auditing and freezing, as well as the fixing of a random seed to ensure exact reproducibility of the clustering partitions.

In parallel, preprocessing was refined through the mathematical characterization of missing data and selective pruning of outliers. However, the main improvement in validation came from two analytical fronts: the integration of inter-cluster inferential tests to confirm the significance of epidemiological differences, and the design of a rigorous sensitivity analysis to test the geometric stability of the solution under data perturbations.

## 7 Recommendations

Accordingly, the present findings should be interpreted as methodological evidence supporting future research rather than as evidence for immediate integration into routine public health surveillance systems.

First, the incorporation of unsupervised machine learning techniques as complementary, expert-supervised tools within epidemiological surveillance platforms merits further exploration. The performance documented by coupling FAMD with K-Means provides preliminary evidence that this approach can identify consistent epidemiological profiles within the analyzed national repository. These findings suggest that unsupervised machine learning may represent a promising complementary analytical approach for exploring analyses and database auditing. However, additional validation is required before its usefulness in operational surveillance environments can be established. It should be emphasized that this framework was validated on a single national repository and has not yet been tested against independent datasets or reviewed by public health specialists; consequently, its integration into operational surveillance systems would require additional validation, institutional piloting, and expert oversight before informing real-world public health decisions.

However, this modernization of the analytical ecosystem also requires strengthening the standardization of epidemiological reporting processes in official notifications. The pronounced geographic and temporal asymmetries observed between clusters highlight the urgency of harmonizing data entry criteria for clinical and epidemiological information, an essential quality-control measure to reduce statistical noise, improve internal consistency, and ensure comparability of data across regions and time windows.

Furthermore, the spatial and temporal heterogeneity observed across the identified clusters suggests that systematic monitoring of epidemiological patterns by department, year, and epidemiological week may provide additional analytical insights when combined with conventional surveillance practices. Although the present study was not designed to evaluate operational surveillance performance or early warning capabilities, the identified profiles indicate that dimensionality reduction and clustering techniques may help reveal latent epidemiological structures deserving of further investigation.

Rather than replacing established descriptive surveillance methods, these approaches could be explored as complementary analytical resources for hypothesis generation and retrospective epidemiological assessments. Their potential contribution to operational public health activities should be evaluated through prospective studies, external validation, and expert assessment before considering routine implementation.

This methodological refinement should proceed in parallel with the exploration and engineering of new epidemiological variables, incorporating environmental factors, macro-climatic records, socioeconomic metrics, entomological censuses, and indicators of local healthcare infrastructure and coverage—determinants that are critical for achieving a more holistic characterization of the phenomenon.

From this perspective of methodological development, future studies may explore the applicability of this workflow to other neglected tropical diseases, vector-borne viral infections, or emerging infectious diseases supported by structured surveillance repositories. Such extensions should be accompanied by disease-specific validation to determine whether similar clustering structures can be reproduced across different epidemiological contexts.

## Data Availability

The raw data supporting the conclusions of this article will be made available by the authors, without undue reservation.
